# LATCHES – a memory aide for the principles of attachment for effective breastfeeding: findings of a regional pilot in the Northeast of England and North Cumbria

**DOI:** 10.1186/s13006-024-00663-8

**Published:** 2024-08-15

**Authors:** Lynette Harland Shotton, Cheryl Elliot, Roslyn Nunn, Kathryn Lane

**Affiliations:** 1 Northumbria University, Newcastle Upon Tyne, UK; 2 North East and North Cumbria Integrated Care Board, Middlesbrough, UK; 3 Darlington Community and Children’s Directorate, The Beehive, Lingfield Point, Darlington, UK

**Keywords:** Breastfeeding, Attachment, Memory aide, Mnemonic, CHINS, LATCHES, Pilot, Northeast England, North Cumbria, England

## Abstract

**Background:**

This paper outlines a pilot of a new memory aide for breastfeeding conducted in the Northeast of England and North Cumbria between April and August 2023. The United Kingdom has some of the lowest rates of breastfeeding, particularly in the Northeast of England, and as such more needs to be done to support mothers to breastfeed for as long as they would like to. Good support from health professionals can be effective in influencing decisions to breastfeed as well as helping to ensure initiation and continuation of breastfeeding but there is evidence to suggest that professionals and students do not always feel adequately trained and it is here, where memory aides may have value.

**Methods:**

Key breastfeeding practitioners and educators were brought together to select one of two memory aides for principles of attachment for effective breastfeeding. The selected memory aide, LATCHS, was piloted with 57 participants with a key role in promotion and support of breastfeeding in the Northeast of England and North Cumbria.

**Results:**

Participants conveyed mixed views about the proposed memory aide with more experienced staff reporting more favourable opinions than student midwives and early years practitioners. Experienced staff felt the new memory aide would complement an early memory aide, CHINS, which focused on principles of positioning.

**Discussion:**

Findings of the pilot indicate there is a role for a mnemonic to help practitioners understand, recall, and retain theory around attachment for effective breastfeeding and that memory aides can play an important role in complementing existing approaches to education and practice. The participants felt the proposed memory aide had some limitations and suggested important ways for it to be improved, particularly in adding an E to reflect the expecting wording. This produced the final memory aide: LATCHES.

**Conclusion:**

Using data from the pilot, the memory aide was refined, and the final version LATCHES agreed for wider dissemination. Future research is needed to understand the value of LATCHES on the wider breastfeeding workforce and whether any future improvements can be made to enhance its utility.

**Supplementary Information:**

The online version contains supplementary material available at 10.1186/s13006-024-00663-8.

## Background

This paper presents an overview and findings of a regional pilot of a new memory aide for breastfeeding that was conducted in the Northeast of England and North Cumbria between March and August 2023.

Global breastfeeding rates have increased by 10% [1] which, given the physical and mental health benefits for mother and baby [2], as well as the reduced costs of healthcare intervention [3] is positive. However, there is great variation in breastfeeding rates between countries, and the United Kingdom (UK) has one of the lowest rates in Europe with only 34% of babies receiving some breastmilk at 6 months. The UK compares badly with other international countries, such as the United States where the rate is 49% [4]. In the UK prevalence is particularly low among very young mothers and in areas where there are high levels of socio-economic deprivation [4]. Indeed, in the Northeast of England, where this pilot was conducted, initiation rates of breastfeeding are much lower than the England average of 74.5% at 59% and at 6–8 weeks this drops significantly with rates across the region varying from 26 to 45% compared to the England average of 42.7% [5]. The variation in breastfeeding rates throughout England is noted in national health care policies such as the NHS Long Term Plan, alongside emphasis on improving the knowledge and skill of the breastfeeding workforce via a range of measures which include UNICEF Baby Friendly Accreditation of key services such as maternity units [6].

It is accepted that good support from health professionals can be effective in influencing both decisions to breastfeed as well as helping to ensure initiation and continuation of breastfeeding but there is evidence to suggest that professionals and students do not always feel adequately trained [7]. The UNICEF Baby Friendly (UNICEF BFI) accreditation programme was established in 1992 and first introduced to the UK in 1994 as a way of ensuring the breastfeeding workforce were equipped with the requisite knowledge and skills to promote and support breastfeeding and is both recognised and recommended in numerous government and policy documents [8]. However, a recent audit in the Northeast of England completed by Local Maternity System Public Health Prevention [5] found that while some key services (maternity, health visiting, Children’s Centres, universities) in the area had achieved accreditation, this was not consistent across the region and many services had lapsed accreditation or had never been awarded accreditation, leading to varying levels of knowledge and skill across the Northeast. The audit highlighted those common barriers to achieving UNICEF BFI accreditation included the associated costs for training and dedicated time to invest and lead this work. This is also compounded by wider issues facing the NHS, particularly staff turnover and shortages of nurses, midwives, and health visitors.

In view of this context the Royal College of Nursing (RCN) [9] suggest there is a role for condensed approaches to CPD, which would help ensure staff continue to develop whilst recognising the significant barriers to facilitate this. With reference to breastfeeding, this could include the introduction of shortened training sessions focused on helping staff develop essential skills, alongside access to more in-depth training where possible. Essential skills should include knowledge and ability to assess and supporting positioning and attachment to the breast, as these are considered key in ensuring successful breastfeeding [10]. It is here where memory aides such as CHINS [11], which offers a simple way for professionals to remember, retain and recall the key principles of positioning for effective breastfeeding, would have value (See Table 1).

**Table 1:** An overview of CHINS [10]

**C**lose: babies need to be close to their mother so they can scoop enough breast into their mouths. Ensure both mother and baby’s clothing and hands are not in the way. **H**ead free: when attaching to the breast babies tilt their heads back. This allows the chin to lead as they come to the breast. Even a finger on the back of the baby’s head will restrict this important movement. **I**n line: the baby’s head and body should be in alignment so they do not have to twist their neck, which would make feeding and swallowing difficult.
**N**ose to nipple: with mother’s nipple resting below the baby’s nose, they will begin to root. As the baby tilts their head back, the nipple will slip under their top lip upwards and backwards to rest between the hard and soft palate. Nose to nipple is that starting point for effective attachment. **S**ustainable: mothers need to be comfortable and relaxed and in a position that suits them best.

CHINS has been widely embedded in UNICEF Baby Friendly Training since 2010 and findings of an evaluation of CHINS funded by the Burdett Trust for nursing shows it has had wide impact across the UK and increasingly internationally. The findings were shared via a poster presentation: A Mixed Methods UK Evaluation of the Memory Aide CHINS at the UNICEF UK Baby Friendly Initiative Annual Conference 22–23 November 2023 [12] (see Additional file 1) and further outputs will be housed via the Northumbria University Knowledge Bank [13]. Feedback from professionals involved in the evaluation of CHINS was overwhelmingly positive about its impact and indicate that CHINS offers a simple way for professionals to remember, retain and recall key theory to promote and support positioning for effective breastfeeding. Many of the participants requested a complementary memory aide for the principles of attachment.

Mnemonics are widely used within health care practice and a recent systematic review conducted by Maheshwari and Kaure refers to their use in helping practitioners remember key theory and recall this to structure and guide practice [14]. Other examples, including El Hussein and Jakubec’s work [15], refer to the value of mnemonics in guiding nursing care plans and Rosenberg et al. [16] outline the role of mnemonics in structuring patient handover information. The focus of this paper is to present the findings of a pilot of a new memory aide for the principles of attachment, and as such, it is important to acknowledge earlier work in this area. In 1994 Jensen, Wallace and Kelsay [17] developed an acronym: LATCH, which was designed to provide a systematic charting system to help practitioners gather information about individual breastfeeding observations. The letter L referred to how well the infant latches onto the breast. The letter A was for the amount of audible swallowing heard. T was for the mother’s nipple type. C was to assess the mother’s level of comfort and H was for the level of help the mother needed to hold her infant to the breast. Arguably, their memory aide provides an important approach to guide the systematic assessment of breastfeeding, however, the extent to which it is used in breastfeeding education and practice in the UK is not known. The authors of this paper could not find any information about this on the UNICEF Baby Friendly Initiative webpages and indeed, the participants in the UK evaluation of CHINS were not aware of such a memory aide, hence their request for one. Consequently, the remainder of this paper focuses on a pilot of a new memory aide for the principles of attachment for breastfeeding.

### Aim of the pilot

The aim of this pilot study was to select one of the two memory aides developed by the first author, shown in Table 2., to pilot in the Northeast of England and North Cumbria to determine whether the memory aide is suitable for sharing more widely with the breastfeeding workforce. The memory aides in this pilot differ from the earlier acronym LATCH in that they draw specifically on theory outlined by the UNICEF Baby Friendly Initiative and aim to provide specific detail about key principles of attachment for effective breastfeeding. Further to this, the nature of this pilot focused on working in collaboration with key breastfeeding practitioners to shape and refine the memory aide to enhance its utility.

**Table 2:** Potential memory aides for attachment

**Memory aide 1: CLASP Principles** *© Lynette Shotton Northumbria University 2023* **C**hin: The chin will lead and when attached baby’s chin should be firmly touching / indenting the breast. **L***arge*: A large / wide gape allows a mouthful of breast tissue to be taken into baby’s mouth. This can be encouraged by the mother gently rubbing her nipple across the baby’s top lip and ensures as much breast tissue as possible below the nipple is taken into baby’s mouth. This is known as asymmetrical attachment. **A***symmetrical attachment /***A***reola*: It is important as it ensures the nipple reaches far back into the mouth as opposed to hitting the hard palate. If visible more areola will be seen above baby’s top lip (this is not always visible). **S***wallowing*: Should be seen and heard and baby’s cheeks should be full and rounded. **P***ain*: Breastfeeding should not be painful.
**Memory aide 2: LATCHS Principles** *© Lynette Shotton Northumbria University 2023* **L -** Large: A large mouthful of breast tissue should be taken into baby’s mouth with as much breast tissue as possible below the nipple. This can be encouraged by the mother gently rubbing the nipple above the baby’s top lip. **A-A**symmetric attachment / **a**reola: This is important as it ensures the nipple reaches as far back into the mouth as opposed to hitting the hard palate. **T**-Top Lip: If visible, more areola will be seen above baby’s **t**op lip (this is not always visible). **C -C**hin and **C**heeks: The **c**hin will lead and when attached baby’s **c**hin should be firmly touching / indenting the breast and baby’s **c**heeks should be full and rounded. **H- H**urting: Breastfeeding should not be painful, and nipples should be the same shape at the end of the feed as at the start. **S**-**S**ucking and **S**wallowing: Baby will take deep rhythmic **s**ucks and **s**wallowing should be heard.

## Methods

The lead author collaborated with two midwifery academics from Northumbria University, a Specialist Health Visitor for Infant Feeding, and a Specialist Lead for Infant Feeding to conduct the pilot project. The collaborators were experienced practitioners who had all completed UNICEF Baby Friendly Initiative Training as well as being experienced in delivering it within their professional practice. The collaborators independently and then jointly critically reviewed both potential memory aides to select one to take forward to pilot across the Northeast of England and North Cumbria. Both memory aides were developed using UNICEF Baby Friendly Initiative theory [18] to ensure they reflected contemporary best evidence and practice. There was consensus that the second memory aide, LATCHS, was the best and was, therefore, taken forward to pilot. Prior to commencing the pilot ethical approval was obtained from Northumbria University: Ethical approval - Project No. 3577.

### Structure and content of the pilot

Between the end of March and June 2023, the collaborators were asked to share LATCHS as widely as possible during breastfeeding education sessions delivered as part of their role. They were asked to show the memory aide and provide an explanation of each of the first letters and how they linked to relevant breastfeeding theory, as outlined in Table 2, before asking participants to take some time to review the information and then participate in focus group discussion to evaluate the memory aide.

The focus group discussion was guided by the following key question:

- What are your first impressions of LATCHS?

Follow up prompts asked:

- Can you think of any limitations of LATCHS?
- If there are limitations, how might they be addressed?
- Do you have any other feedback?

### Study site and participants

The study site covered the Northeast and North Cumbria, and this was largely for practical reasons given that the collaborators involved in the pilot covered this geographic area in their professional roles. At the end of breastfeeding education sessions, the collaborators asked the participants if they wished to take part in the pilot. Information about the pilot was shared and participation was based on informed choice. Consent was obtained verbally and in writing and given their professional and student status, all participants were over 18 years of age and deemed able to provide consent. The focus groups were audio recorded and participants were anonymised.

In total there were 57 participants from a range of professional and non-professional backgrounds (see Table 3). The size of the sample meant that extensive feedback about LATCHES was obtained from a range of practitioners all involved in the promotion and support of breastfeeding. To protect anonymity the letter R and a number between 1 and 57 were assigned to each of the 57 participants.

**Table 3:** Professional background of participants

Professional Role	Number of participants
Infant Feeding Leads from midwifery, health visiting and Family Hubs (Family Hubs are local support centres for families)	23
Master’s Level midwifery Students	9
University midwifery lecturers	6
Early Years Practitioners (Early Years Practitioners work closely with babies, toddlers and pre-school children, their families and / or carers to support physical, educational, and emotional development. In the UK they often work in health services as part of an inter-disciplinary team).	6
Specialist Public Health Midwives	5
Neonatal Infant Feeding Leads	3
Infant Feeding Maternity Support Workers	3
Neonatal Care Co-ordinators	2

### Data analysis

Focus group recordings were transcribed verbatim and analysed by the lead author using a deductive approach based on the evaluation questions. The overarching themes were support for LATCHS and limitations of LATCHS and areas for improvement. Initial analysis was undertaken by the first author and then scrutinised and final themes agreed in collaboration with the co-authors.

## Results

### Support for LATCHS

The participants felt that there was a lot to learn and remember about breastfeeding and that it was important to “get it right” (R6, Infant Feeding Lead) to ensure mothers and their babies received the best possible care and did not give up breastfeeding before they wished to. Consequently, there was consensus that a memory aide for the principles of attachment would be a welcome intervention. This was largely based on the sustained success of CHINS, which participants were all aware of and had used in their practice and the fact there was currently no standard mechanism to remember principles of attachment. One participant indicated:

“I was a community midwife before I was in this role and CHINS I used all day, every day, and I absolutely loved it and so did my colleagues. So, this one, I’ll remember that now” (R10, Infant Feeding Lead).

Respondents felt that CHINS had helped with their practice because it was simple and easy to use, and one participant felt that LATCHS “accompanies CHINS beautifully” (R1, Infant Feeding Lead).

Several of the participants liked the overall structure of LATCHS and the fact that it made sense to them. For example,

“But I think the way it’s laid out and it’s, it’s relevant that title, isn’t it to you know, latching the baby fixing on and things” (R11, Infant Feeding Lead).

However, this was not the case for all participants and generally, LATCHS was more positively received by professional registrants, especially those with a key responsibility or role for delivering breastfeeding education.

### Limitations of LATCHS and areas for improvement

Those from an Early Years Practitioner background were less supportive of LATCHS and several felt that in comparison to CHINS, which they liked, and were used to using, LATCHS would be more difficult to remember. Both early years practitioners and midwifery students debated at length the term a-symmetric attachment and felt that areola would be sufficient. They also felt that T for top lip could be confusing, as the mother herself would not be able to see this. The use of H for hurting was also questioned, as illustrated in the following quote:

“The word hurting is a negative word […] this may make mothers think breastfeeding is always painful” (R56, Early Years Practitioner).

There was also discussion about the fact that LATCHS did not match the dictionary spelling of the word latches and that this might make it more difficult to remember. One participant suggested:

“I would maybe spell it LATCHES […to avoid] trying to remember and “e” that doesn’t exist” (R51, Master’s Midwifery Student).

## Discussion

The participants in this pilot were very aware of the importance of promoting and supporting breastfeeding and their professional duty in providing high quality and evidence-based practice. Arguably this reflects the context presented at the beginning of this paper, where there is increasing recognition that breastfeeding rates in England compare less favourably than other countries [4], alongside awareness and commitment at strategic level to drive forward measures to improve breastfeeding rates by improving education, skills, and practice [6]. Despite this, the participants were aware of the challenges faced by practitioners in delivering high quality breastfeeding support and this linked to both the context of busy professional roles with competing demands on time and the need for a wide-ranging skillset but also, as outlined by (R6) recognition that there is “a lot to learn” to provide high quality breastfeeding support.

The memory aide for CHINS and the new memory aide in this pilot focus on the two key areas of importance for effective breastfeeding: positioning and attachment. These are often considered critical to effective breastfeeding, and it is suggested that in the event of difficulty with breastfeeding these should be assessed as part of the diagnostic process [19]. Whilst the ambition for key services to achieve UNICEF Baby Friendly Accreditation is positive, it must be accepted that this will take time to achieve, particularly given the varying rates of accreditation across the UK [6] and regionally [5]. In view of this there is a strong argument for simpler approaches to supporting the development of key skills [8], which could be introduced quickly and widely across the breastfeeding workforce as a measure to address the skills gap and in the absence of more widespread UNICEF Baby Friendly Training. It is here where these two memory aides could add value and help practitioners develop skills in these two important areas.

Whilst it is suggested that mnemonics are often more beneficial to students and those learning new materials [14], it is interesting in this pilot that LATCHS was viewed more negatively by student midwives and early years practitioners. However, as identified previously, this is perhaps because in its original form LATCHS made less sense to them because it did not sound the way they would expect it to be spelled and, as such did not help them to organise the theory in a meaningful way. Those practitioners with more experience may have been able to understand how LATCHS could support their already established practice and therefore, even in this form could see how they would use it. This finding demonstrates the importance of conducting pilot work to test interventions and use the data to both identify problems in design and utility as well as to make enhancements [20]. This pilot was particularly helpful in doing that and as outlined, the participants were able to provide a robust critique of LATCHS, as well as helpful suggestions about how it could be enhanced to improve its utility in practice. This resulted in amendments which produced the final memory aide: LATCHES, which includes the addition of the E to ensure it is spelled the way it sounds. Table 4 provides an overview of LATCHES and the explanation of the theory behind each of the letters.

**Table 4:** LATCHES: a memory aide for the principles of attachment for effective breastfeeding. *© Lynette Shotton Northumbria University 2023*

**L** *for large gape.* Look for the baby’s mouth to open widely. Mother should move baby to her breast with the baby’s head tilted back and the chin leading. Baby’s tongue will move down and forward so the baby can scoop a **l**arge mouthful of breast with the nipple aimed towards the rear roof of baby’s mouth. **A***for areola and***T***for top lip*: If visible, more **a**reola will be seen above the baby’s **t**op lip. This will result in a-symmetric attachment. **C***for chin and cheeks*: Baby’s chin leads and will indent the breast and baby’s cheeks will be full and rounded.
**H** *for how does it feel?* Check **h**ow it feels for mother and ensure feeding is comfortable. **E***for***e***xamine and***e***xplore*: Conduct a thorough **e**xamination, which will include a breastfeeding assessment and then **e**xplore future support needs. **S***for***s***ucking and***s***wallowing*: Look and listen for **s**ucking and **s**wallowing but remember, these will be appropriate to the age of the baby.

## Conclusion

This regional pilot of this new memory aide to help breastfeeding practitioners understand, retain, and recall principles of attachment for effective breastfeeding underlines the value of working in collaboration with key stakeholders and participants to improve interventions designed to support effective breastfeeding practice. It is accepted that memory aides such as this may not work for everyone but equally that they can play a key role in improving practice and ultimately helping mothers to breastfeed their babies for as long as they would like to.

The aim of both CHINS and LATCHES is not to replace existing approaches to the education and training of the breastfeeding workforce, or to replace existing breastfeeding assessment tools. Instead, it is hoped that they may play a complementary role and add another approach to ensuring practitioners have the requisite skills to promote and support breastfeeding. Within the United Kingdom, CHINS has been incorporated into the Practical Skills Review Form – Breast Feeding and Hand Expression [18] which suggests that simple memory aides such as this have utility in helping effective breastfeeding assessment.

By sharing LATCHES more widely with breastfeeding practitioners and educators, they will be able to determine whether this supports their retention and use of evidence-based theory to guide their practice. In doing so, this will pave the way for a future evaluation of LATCHES to understand how it has been received by the breastfeeding workforce as well as to identify whether and how it can be improved.

### Electronic supplementary material

Below is the link to the electronic supplementary material.

Supplementary Material 1

## Data Availability

The datasets used and/or analysed during the current study are available from the corresponding author on reasonable request.
