# Supplementary material for: LATCHES – a memory aide for the principles of attachment for effective breastfeeding: findings of a regional pilot in the Northeast of England and North Cumbria
Source: Int Breastfeed J. 2024 Aug 15;19:57. doi: 10.1186/s13006-024-00663-8 (PMC11325656; doi:10.1186/s13006-024-00663-8)
Supplement: Supplementary file 1 — Supplementary Material 1 [file 13006_2024_663_MOESM1_ESM.pdf]

UNITED KINGDOM

THE BABY FRIENDLY INITIATIVE

A mixed methods evaluation of the memory aide for positioning: CHINS

This evaluation was funded by the Burdett Trust for Nursing

Dr Lynette Shotton
lynette.shotton@northumbria.ac.uk

Introduction

This poster reports the findings of a mixed methods evaluation of CHINS. CHINS was developed in 2010 (Harland, 2011) to help practitioners remember, recall and use theory to support positioning for effective breastfeeding. CHINS has been adopted by the UNICEF Baby Friendly Initiative and is used widely in healthcare practice, but until now has not been subject to formal evaluation of impact in practice.

1

CHINS

Close: babies need to be close to their mother so they can scoop enough breast into their mouths. Ensure both mother and baby's clothing and hands are not in the way.
Head free: when attaching to the breast babies tilt their heads back. This allows the chin to lead as they come to the breast. Even a finger on the back of the baby's head will restrict this important movement.
In line: the baby's head and body should be in alignment so th`ey do not have to twist their neck, which would make feeding and swallowing difficult.
Nose to nipple: with mother's nipple resting below the baby's nose, they will begin to root. As the baby tilts their head back, the nipple will slip under their top lip upwards and backwards to rest between the hard and soft palate. Nose to nipple is that starting point for effective attachment.
Sustainable: mothers need to be comfortable and relaxed and in a position that suits them best.

2

Theory

The four constructs of Normalisation Process Theory (May et al. 2015),Coherence; Cognitive Participation, Collective Action and Reflexive Monitoring were used to develop survey and focus group questions and to analyse the data.

3

Methods

Ethical approval was obtained from Northumbria University study ID 40808.
A flier was distributed via breastfeeding, professional networks and social media to purposively recruit breastfeeding practitioners across the United Kingdom (UK).
Phase 1 115 practitioners from across the UK completed an online survey
Phase 2 16 survey respondents took part in five focus groups
Data analysis Survey data was analysed using bivariate and multivariate tests. Focus group data was analysed using the four constructs of Normalisation Process Theory

4

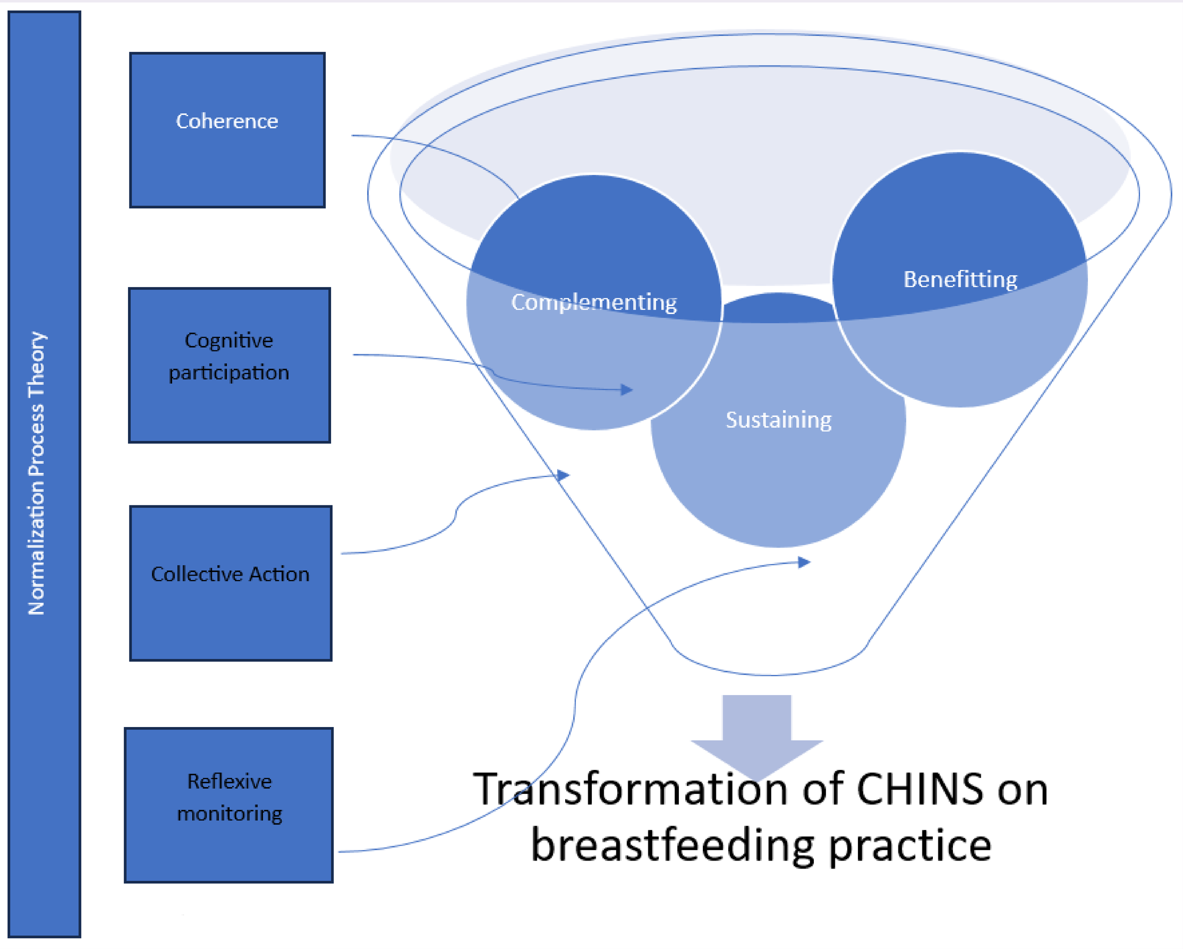

Findings

Findings from the survey and focus groups were integrated and aligned to the four constructs of normalisation process theory to produce the following schema:

Complementing

UNICEF played a key role in the normalization process. **Survey findings** showed that practitioners were five times more likely to value CHINS if they had completed UNICEF Baby Friendly Initiative Training. This was echoed in focus group findings where most of the participants made a connection between UNICEF training and CHINS. For example, one participant indicated:

"That's all I've ever used to teach breastfeeding, because that's the way I was taught. So, first and foremost, I would teach [CHINS]– if it fits with current evidence. Which… Yeah, there's nothing saying anything has really changed there. So, I would use that. Probably we use it alongside the fact that UNICEF are really established in wanting to use that. And, I mean, that is something, when you go to the study day, that they do. It's in their slides, and they do use the CHIN mnemonic." (R7).

The participants also cited key resources and guidance, which helped to promote use of CHINS. These included the Practical Skills Review (UNICEF BFI), The Mothers and Others Guide, as well as local NHS service level guidance and in mobile apps:

"We have a similar guide…An Essential Guide to Feeding Your Baby and we have CHINS in there and we have it on the wall, we have an App, and it really links to UNICEF. So, it's (CHINS) all on the app as well" (R11).

So, here, there was clear evidence of normalisation but the reason behind this is outlined in **survey findings** which showed professional duty in supporting breastfeeding was associated with practitioners driving the use of CHINS and feeling that they had had sufficient training and that use of CHINS was supported by management. **Focus group** data supported this:

"The Scottish government has got to change the drop off at six to eight weeks, then, by 2025.[..] But I use CHINS every day.[..] We, sort of, adopted it… It came through UNICEF, obviously, for us as well, as a Trust. As a board. So, all our staff have UNICEF training, you know, as the requirements. As part of the BFI initiative. So, it just fed through… Through the training. And I… We use it because we know it helps mums to remember." (R14)

"And I think now there's such a push, isn't there, with the NHS Long-term Plan that all maternity services need to be accredited. That it is… It is [CHINS] becoming more widely shared in the training". (R15).

Benefiting practitioners and service users

Survey findings found staff who had had UNICEF BFI training were more likely to value CHINS and this improved their confidence in providing breastfeeding education to students and peers. This was also evident in **focus groups** where practitioners talked about simplicity of CHINS, the beneficial structure and perceived benefits to both practitioners and mothers:

I think obviously CHINS… With it being so simple, it's given people the confidence to, kind of, discuss, you know, the principles of positioning with parents. So, even if you are, you know, very new to the NHS, very new with supporting with infant feeding… Just being, kind of, taught that mnemonic. It does – it kind of gives the confidence to then pass that message to the mum. So, I don't think you have to be an expert in infant feeding to… To apply the CHINS principles. I think it works across the board. (R1)

So, it's (CHINS) just used far and wide. And if you speak to women, once you've taught them it once it's really easy for them (R7)

I suppose I would class myself as an expert, but it's just nice with a busy clinic of 31 mums, that you know, I just have to remember CHINS... and I don't have to think oh my God "(R12).

There was also evidence of CHINS being used to challenge poor practice:

"Even on NHS images – it looks so lovely and snuggly, but the head isn't free. And that's my reference. I say, oh, look at this – this isn't CHINS (R13)

And to highlight in professional records when they had delivered good care:

"and then you've documented it (CHINS)– because, actually that's what you have done" (R16)

Sustaining CHINS

Survey findings showed that if length of practice was more than 10 years, staff were more confident in providing breastfeeding education to students, peers and service users. Focus group findings supported this and revealed that CHINS helped experienced staff as well as new staff:

"I suppose I would class myself as an expert, but it's just nice with a busy clinic of 31 mums, that you know, I just have to remember CHINS... and I don't have to think oh my God "(R12).

"And we've recently trained some staff that have had no, sort of, training before. So, completely new to feeding. And the comments from them have been very much like they just forget the principles, completely, when they were learning this themselves. So, actually, using the CHIN acronym just really, really helps them to actually remember themselves what they're going through with the women" (R1)

However, staff were aware of attrition and skill mix, as identified by one focus group participant:

"In the NHS, the turnaround of staff is just so huge and so continuous. So, you depend on, kind of, having a regular reminder training sessions, which aren't always facilitated when services are so busy. In… In my department, we provide an infant feeding update every three years, and it's part of that update. But three years is a long time" (R15)

Transformation of practice

Key focus group findings indicated the widespread use of CHINS had had a transformational impact, in providing structure, standardisation, and addressing outdated approaches. For example:

"I think staff liked tummy to mummy, because it sort of rhymed. But then UNICEF said, well, it isn't… that doesn't work for everybody, but then that's hard to, sort of, say. So, CHINS just makes it… Because it's your own boobs, isn't it? It's the own size of your baby, and your own size of your boobs, really, that..." (R11)

"Because it can be used from anybody that's supporting breastfeeding. And it is literally the go to. So, yeah, you're saying it is a small thing, but I think it's massive. I do think it's massive". (R4)

"you've not invented a new drug or a special medicine for making… But it's as effective as that. That's what it is. It's a… As you say, it's a communication tool for breastfeeding" (R13)

One survey participant had a negative view of CHINS and felt it was too simple and produced problems in practice. However, this was not the norm and indeed one focus group participant responded to this:

"No, definitely not. I think just… The only time where it can, kind of, cause frustration for mothers, is if they've been told something entirely different previously. And I'm coming in and saying, right, this is an approach that I would recommend. Someone told me something entirely the opposite… But that's not the fault of the abbreviation, that's just what's happened previously". (R15)

Implications

- UNICEF BFI training has been pivotal in producing widespread awareness and use of CHINS and plays an important role in sustaining its use
- Further dissemination and evaluation with a larger sample would help confirm the impact of CHINS
- A similar memory aide for attachment may have similar value.
- Consideration must be given to developing and sustaining skills of the breastfeeding workforce.

Want to know more about my work?
Visit the CHINS and Attachment area of the Northumbria University Knowledge Bank. Access via QR or link below:
https://www.northumbria.ac.uk/business-services/engage-with-us/research/ip-and-commercialisation/knowledge-bank/

References

Harland, L. (2011) Remember: CHIN, Community Practitioner, 84 (1), pp. 18.
May, C., Rapley, T., Mair, F.S., Treweek, S., Murray, E., Ballini, L., Macfarlane, L., Girling, M. and Finch, T.L. (2015) Normalization Process Theory On-line Users' Manual, Toolking and NoMAD Instrument. Available from http://www.normalizationprocess.org (Accessed 7.7.23)
